# *Serendipita indica* Enhances Drought Tolerance in *Phoebe sheareri* Seedlings by Improving Photosynthetic Efficiency, Stimulating the Antioxidant Defense System, and Modulating Hormone Synthesis

**DOI:** 10.3390/jof11100717

**Published:** 2025-10-03

**Authors:** Xiaohu Chen, Rui Sun, Die Hu, Yujie Yang, Zihan Cheng, Ping Hu, Yongjun Fei

**Affiliations:** 1 Hubei Key Laboratory of Spices & Horticultural Plant Germplasm Innovation & Utilization, Germplasm Resources Evaluation and Innovation Center of *Phoebe* Nees and *Machilus* Nees, College of Horticulture and Gardening, Yangtze University, Jingzhou 434025, China; 2023720987@yangtzeu.edu.cn (X.C.); 2024721002@yangtzeu.edu.cn (R.S.); hudie.16@163.com (D.H.); yjyang@yangtzeu.edu.cn (Y.Y.); zihancheng@yangtzeu.edu.cn (Z.C.); 2 School of Architecture, Hubei Engineering University, Xiaogan 432100, China; huping_2001@163.com

**Keywords:** *Serendipita indica*, *Phoebe sheareri*, drought resistance, photosynthetic efficiency, oxidative damage, hormones

## Abstract

In the context of contemporary climate change, drought is widely recognized as a major stressor affecting plant growth. While numerous studies have demonstrated that *Serendipita indica* enhances stress resistance in host plants and is widely used in agriculture, research on its symbiotic interactions with woody plants for improving drought tolerance remains limited. This study investigated the effects of *S. indica* inoculation on the growth of *Phoebe sheareri* seedlings under varying drought conditions—well-watered (WW), moderate drought (MD), and severe drought (SD)—and explored the physiological mechanisms underlying improved drought resistance. The results showed that under WW conditions, *S. indica* inoculation promoted seedling growth and development. Under MD and SD conditions, although drought stress inhibited growth, inoculation significantly increased plant biomass, root parameters, chlorophyll content, and photosynthetic efficiency. Additionally, it alleviated drought-induced damage by reducing REC, MDA, H_2_O_2_, and O_2_^−^ levels, while enhancing SOD, POD, and CAT activities, and increasing root ABA, GA, IAA, and CTK content. Under MD stress, adaptive changes in root architecture and hormone levels were observed, including increases in total root length, surface area, volume, average diameter, and elevated IAA and CTK levels—all of which were further enhanced by *S. indica* inoculation. In conclusion, symbiosis with *S. indica* improved drought tolerance in *P. sheareri* seedlings likely through enhanced photosynthesis, antioxidant enzyme activity, and hormone regulation.

## 1. Introduction

Global climate change is reshaping the Earth’s ecosystems at an unprecedented rate and intensity. Among these changes, drought has emerged as one of the most destructive abiotic stress factors, with significant increases in its frequency, duration, and geographical distribution [1,2]. Drought not only directly leads to water scarcity but also profoundly affects the structure, function, and stability of terrestrial ecosystems [3]. In forest ecosystems, drought is a key driver of forest decline, biodiversity loss, and the degradation of ecosystem services, often resulting in tree wilting, growth inhibition, and even large-scale mortality [4]. It is particularly noteworthy that the seedling stage—the most vulnerable and regeneration-critical phase in the life history of woody plants—is highly sensitive to water stress [5]. Therefore, gaining a deeper understanding of the mechanisms through which drought stress affects seedlings, and exploring effective mitigation strategies, are of great significance for safeguarding forest resources and supporting ecological restoration.

*Phoebe sheareri* is a precious tree species endemic to China, belonging to the genus *Phoebe* within the family Lauraceae [5]. It exhibits outstanding ecological value as a keystone constructive species in subtropical evergreen broad-leaved forests. With a well-developed root system and dense canopy, it plays a significant role in water conservation and soil retention, while providing essential habitats for birds, insects, and epiphytic plants, making it an excellent species for ecological restoration and urban greening [6]. The species also possesses considerable economic and cultural importance: its wood is hard, corrosion-resistant, and displays an elegant texture, making it suitable for high-end furniture, carvings, and construction, thereby commanding high market value [7]. Furthermore, the roots and leaves of *P. sheareri* can be used medicinally for eliminating dampness, reducing swelling, promoting blood circulation, and alleviating pain. The leaves and fruits contain natural bioactive compounds from which essential oils can be extracted, demonstrating potential for pharmaceutical and industrial applications [8,9]. However, the seedlings of *P. sheareri* are characterized by shallow root systems and high transpiration rates, making them highly susceptible to stress under combined seasonal drought and climate change, resulting in reduced biomass accumulation, declined photosynthetic capacity, and increased mortality rates, which severely limits both natural regeneration and artificial afforestation efforts [10].

*Serendipita indica* is a root endophytic fungus initially isolated from the rhizosphere soil of desert shrubs in the Thar Desert, India [11]. It colonizes the inner cells of plant root tissues, where it acquires necessary nutrients from the host while simultaneously enhancing the plant’s nutrient uptake. The extensive hyphal network of *S. indica* extends into the soil, facilitating the acquisition of phosphorus, nitrogen, and other essential elements, thereby improving soil nutrient utilization and establishing a mutualistic symbiotic relationship with the plant [12,13,14]. Numerous studies have demonstrated that inoculation with *S. indica* exerts significant positive effects on plant growth and stress resistance. Research has confirmed that colonization by *S. indica* notably increases relative water content and promotes the accumulation of soluble proteins and proline. It also modulates the synthesis of hormones such as IAA, JA, and ABA, thereby stimulating morphological adaptations in the root system—including increased total root surface area, root volume, and root diameter—which collectively enhance water acquisition under drought conditions [15,16,17]. Furthermore, *S. indica* activates the plant’s antioxidant enzyme system, including superoxide dismutase (SOD), peroxidase (POD), and catalase (CAT). The enhanced activity of these enzymes helps maintain biomembrane integrity, regulate osmotic balance, mitigate membrane lipid peroxidation damage caused by drought, and inhibit the accumulation of reactive oxygen species (ROS) [18,19,20]. Although primarily colonizing the roots, *S. indica* also positively influences leaf photosynthesis. It delays leaf rolling under drought stress, enhances the production of photosynthetic pigments, and helps sustain a high photosynthetic rate, thereby improving the plant’s overall adaptability to arid environments [21,22]. However, to date, most studies on the growth-promoting and stress-resistant effects of *S. indica* have focused on herbaceous plants and crops, with research on its symbiotic relationships with woody plants remaining limited.

Therefore, this study investigates the physio-ecological mechanisms by which *S. indica* inoculation enhances drought tolerance in *P. sheareri* seedlings. We aimed to (1) confirm the formation of a functional symbiosis, and (2) assess its mitigating effects on key physiological processes—including growth, photosynthesis, the antioxidant system, and phytohormone homeostasis—under drought stress. Our findings elucidate the mechanisms behind this fungal-enhanced drought resilience, thereby contributing to the theoretical understanding of woody plant–fungal symbiosis under abiotic stress and providing a practical foundation for using mycorrhizal technology to improve afforestation survival in arid and degraded environments.

## 2. Materials and Methods

### 2.1. Plant Materials and Growth Conditions

The experiment was conducted in a greenhouse at the Comprehensive Practical Teaching Base of the College of Horticulture and Landscape Architecture, Yangtze University, located in Jingzhou City, Hubei Province, China.

Two-year-old seedlings of *P. sheareri*, uniform in growth, healthy, and free from pests and diseases, were used as plant materials. The seedlings were bare-root harvested and transplanted individually into pots filled with field soil. Prior to the experiment, the field soil was sterilized by autoclaving (121 °C, 0.11 MPa, 2 h) to eliminate microbial contaminants.

### 2.2. Fungal Preparation and Inoculation

The fungal strain of *S. indica* used in this experiment was screened and preserved by the Germplasm Resource Evaluation and Innovation Center for *Phoebe* and *Machilus* at Yangtze University. The strain was initially cultured on Pachlewski (PACH) solid medium for two weeks. Subsequently, two mycelial plugs (1 cm in diameter) were transferred into 250 mL of PACH liquid medium and subjected to shaken culture in the dark (120 r·min^−1^, 25 °C) for four weeks. Subsequently, the mycelium was then filtered using 20-mesh gauze and blended with distilled water to prepare a suspension at a concentration of 30 g/L. The resulting mycelium was then homogenized using a homogenizer.

After a two-month seedling acclimatization period, inoculation was performed via root irrigation. Each seedling in the inoculated group received 50 mL of the fungal homogenate applied to the rhizosphere, while control seedlings were treated with an equal volume of sterile blank culture medium. The inoculation was repeated every 7 days for a total duration of two months.

### 2.3. Experimental Design

Seedlings of similar growth status, either inoculated with *S. indica* (+*Si*) or non-inoculated controls (–*Si*), were subjected to three drought treatments: well-watered (WW, 80% field capacity), moderate drought (MD, 50% field capacity), and severe drought (SD, 30% field capacity). Each treatment included 24 inoculated and 24 non-inoculated plants. Soil moisture was maintained daily at the target levels through the gravimetric method at 6:00 PM. Following the 25-day treatment period, all plants (144 seedlings) were destructively sampled for subsequent physiological and biochemical measurements.

### 2.4. Measurements

#### 2.4.1. Determination of Fungal Colonization and Biomass

We placed the cleaned roots in 10% KOH solution, and heated them in a water bath at 90 °C for 60 min. After cooling to room temperature, we discarded the KOH solution, rinse with distilled water 8–10 times, and transferred the roots to a beaker. Subsequently, fungal colonization was assessed using the Trypan blue staining method as described by Yang et al. [23]. One hundred and twenty root segments, each 1 cm in length, were randomly selected and observed under an optical microscope at magnifications of 4× and 10×, with photographs taken accordingly (Olympus CX21, Shanghai, China). The colonization rate was calculated as follows:

Colonization rate (%) = (Number of colonized segments/Total segments observed) × 100%.

After harvest, soil adhering to the roots was carefully rinsed off under running water. The plants were then blotted dry and separated into above-ground and below-ground parts. The samples were first heated at 105 °C for 30 min to deactivate enzymes, followed by drying at 65 °C until a constant weight was achieved. The dry weights of the shoot and root biomass were measured separately.

#### 2.4.2. Measurement of Gas Exchange Parameters

Leaf gas exchange parameters were measured prior to plant sampling. On clear days, between 9:00 AM and 12:00 PM, fully expanded mature leaves (the third from the top of each plant) were selected for measurement. An portable photosynthesis system (LI-COR Environmental, LI-6800, Lincoln, NE, USA) was used to determine key parameters including: Transpiration rate (Tᵣ), Net photosynthetic rate (Pₙ), Intercellular CO_2_ concentration (Cᵢ) and Stomatal conductance (Gₛ).

#### 2.4.3. Measurement of Physiological and Biochemical Indicators

Root morphological parameters, including total root length, surface area, volume, and average root diameter, were analyzed using an EPSON scanner (model v3.771, Epson Co., Ltd., Tokyo, Japan) in conjunction with WinRHIZO Pro 2007a image analysis software.

Leaf relative electrical conductivity (REC) was measured with a LEICI DDB-303A conductivity meter (Shanghai, China) [24]. The contents of hydrogen peroxide (H_2_O_2_) and superoxide anion (O_2_^−^) were determined using a Shimadzu UV-3600 UV-VIS-NIR spectrophotometer (Kyoto, Japan), as outlined by Zhou et al. [25]. The activities of superoxide dismutase (SOD), peroxidase (POD), catalase (CAT), and the concentration of malondialdehyde (MDA) were assessed using commercial assay kits (Nanjing Jiancheng Bioengineering Institute, Nanjing, China).

Endogenous phytohormones in the roots—abscisic acid (ABA), gibberellin (GA_3_), auxin (IAA), and cytokinin (CTK)—were quantified using high-performance liquid chromatography (HPLC; LC-100 system, Yokogawa Electric, Tokyo, Japan) based on the method described by Liu et al. [26]. The HPLC conditions were set in accordance with Wu et al. [21]. All measurements were performed with three biological replicates and three technical replicates per sample.

### 2.5. Data Analysis

We organized our data using Microsoft Excel 2010. A one-way analysis of variance (ANOVA) was performed using SPSS 27.0 (IBM Corp, Armonk, NY, USA). Comparisons were analyzed using Duncan’s multiple range method, and differences were considered statistically significant if the *p*-value was less than 0.05. Graphs and images were processed using Graphpad Prism 9.0 and Adobe Photoshop 2021.

## 3. Results

### 3.1. Colonization of S. indica on the Roots of P. sheareri Seedlings and Its Effects on Plant Growth and Biomass Under Drought Stress

**Figure 1:**
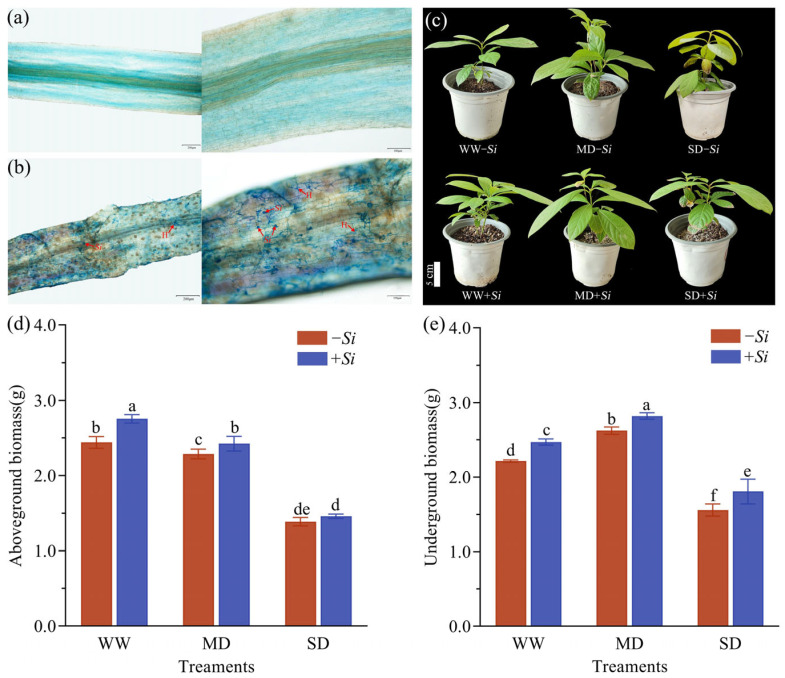
Uncolonized roots (**a**), colonized roots (**b**), and the effects of *S. indica* inoculation on plant morphology (**c**), aboveground biomass (**d**), and underground biomass (**e**) under drought stress. Abbreviations: *Si*—*S. indica*; H—hyphae. The data are presented as the means ± SEs (n = 6); different letters above the bars indicate significant (*p* < 0.05) differences. Abbreviations: WW, well-watered; MD, moderate drought; SD, severe drought; *+Si*, inoculation with *S. indica*; *-Si*, inoculation without *S. indica*.

No colonization was observed in the *-Si* treatment group (Figure 1a). In contrast, spores and mycelium structures were clearly visible in the *+Si* treatment group, indicating that *S. indica* had successfully infected the root system of *P. sheareri* seedlings and formed symbiotic structures (Figure 1b). Among 120 randomly selected root segments from the *+Si* treatment group, 87 were colonized by *S. indica*, resulting in a colonization rate of 72.5%.

Under normal water supply conditions, *P. sheareri* seedlings exhibited an over-all healthy and relaxed state; Under drought stress, leaves in the *-Si* treatment group gradually turned yellow from the leaf tip toward the petiole, showing varying degrees of wrinkling and wilting. The leaf damage became more severe as the drought stress intensified; Under equivalent drought conditions, plants in the *+Si* treatment group exhibited less leaf damage compared to the control group, with milder symptoms of leaf curling, dehydration, yellowing and wilting were milder (Figure 1c).

Both drought stress and *+Si* treatment had significant effects on plant growth (Figure 1d,e). As drought stress intensified, aboveground biomass gradually decreased, while underground biomass first increased and then declined. Compared with the *-Si* treatment, the *+Si* treatment significantly increased aboveground biomass and underground biomass by 18.9% and 11.5%, respectively, under WW conditions; under MD conditions, above-ground biomass and underground biomass increased significantly by 6.0% and 7.5%, respectively; under SD conditions, underground biomass increased significantly by 15.8%, while aboveground biomass showed an increase but did not reach a significant level.

### 3.2. Effects of S. indica on Root Growth of P. sheareri Seedlings Under Drought Stress

**Figure 2:**
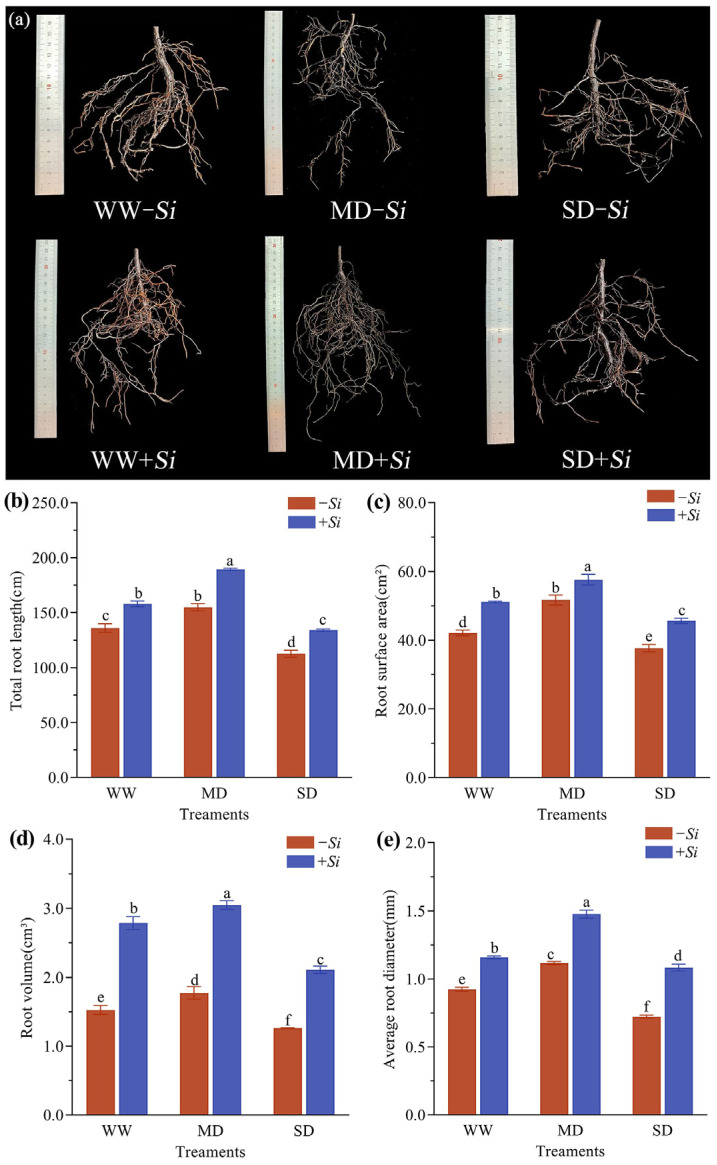
Effects of *S. indica* on root morphology (**a**), total root length (**b**), root surface area (**c**), root volume (**d**), and average root diameter (**e**) under drought stress. The data are presented as the means ± SEs (n = 6); different letters above the bars indicate significant (*p* < 0.05) differences. Abbreviations: WW, well-watered; MD, moderate drought; SD, severe drought; *+Si*, inoculation with *S. indica*; *-Si*, inoculation without *S. indica*.

Under different treatments, the root morphology of *P. sheareri* seedlings showed significant differences (Figure 2a). Under WW treatment, the *-Si* treatment roots had a relatively developed system with longer primary roots but fewer lateral roots. In contrast, the *+Si* treatment significantly promoted the formation of lateral roots and branching structures. Under MD treatment, both primary and lateral roots became longer and thicker, and the *+Si* treatment led to a denser distribution of lateral roots. SD treatment suppressed root growth, resulting in shorter primary roots, sparse lateral roots, and signs of withering and browning. The *+Si* treatment partially alleviated drought stress, manifested by an increase in lateral root number and better-preserved root morphology, thereby reducing drought-induced damage.

As drought stress intensified, the total root length, root surface area, root volume, and average root diameter of *P. sheareri* seedlings all exhibited a trend of initially increasing and then decreasing; Under the same moisture conditions, all the indicators in the *+Si* treatment were significantly improved (Figure 2b–e). Under WW treatment, the *+Si* treatment significantly increased total root length, root surface area, root volume, and average root diameter by 16.2%, 21.4%, 82.7%, and 25.5%, respectively, compared to the *-Si* treatment. Under MD treatment, the corresponding indicators increased significantly by 22.3%, 11.5%, 71.8%, and 32.1%, respectively. Under SD treatment, they still showed significant increases of 19.3%, 21.1%, 67.0%, and 49.5%, respectively.

### 3.3. Effects of S. indica on Chlorophyll Content and Photosynthetic Efficiency in P. sheareri Seedlings Under Drought Stress

**Figure 3:**
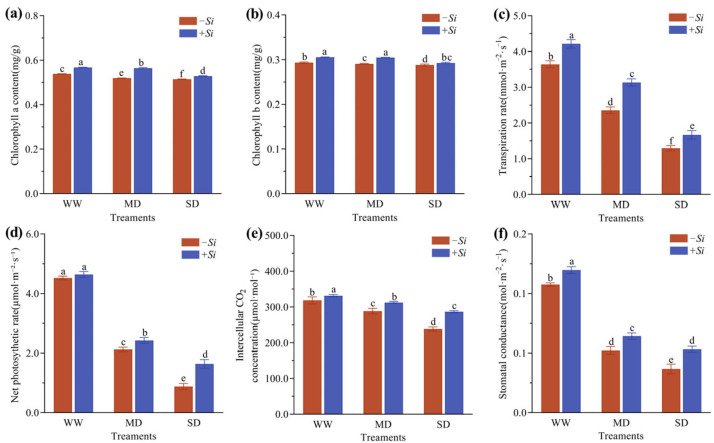
Effects of *S. indica* on chlorophyll a content (**a**), chlorophyll b content (**b**), transpiration rate (**c**), net photosynthetic rate (**d**), intercellular CO_2_ concentration (**e**), and stomatal conductance (**f**) under drought stress. The data are presented as the means ± SEs (n = 3); different letters above the bars indicate significant (*p* < 0.05) differences. Abbreviations: WW, well-watered; MD, moderate drought; SD, severe drought; *+Si*, inoculation with *S. indica*; *-Si*, inoculation without *S. indica*.

As drought stress intensified, both chlorophyll a and chlorophyll b content in *P. sheareri* seedlings showed a gradual decline trend. Under the same drought conditions, the *+Si* treatment significantly increased the Content of Various Chlorophylls (Figure 3a,b). Under WW treatment, chlorophyll a and chlorophyll b in the *+Si*-treated group increased significantly by 5.3% and 4.3%, respectively, compared to the *-Si*-treated group. Under MD treatment, they increased by 8.5% and 4.8%, respectively. Compared to the *-Si*-treated group, the *+Si*-treated group under SD treatment showed significant increases of 2.7% and 1.6%, respectively.

Concurrently, as drought severity intensified, the transpiration rate (Tr), net photosynthetic rate (Pn), intercellular CO_2_ concentration (Ci), and stomatal conductance (Gs) of *P. sheareri* seedlings all exhibited gradual downward trend; Under the same drought conditions, the *+Si* treatment resulted in an increase in all these photosynthetic parameters (Figure 3c–f). Under WW treatment, compared with the *-Si* treatment, the *+Si* treatment significantly increased Tr, Gs, and Ci by 15.8%, 11.3%, and 8.9%, respectively. Under MD treatment, the *+Si* treatment significantly increased Tr, Pn, and Ci by 33.0%, 14.2%, and 8.4%, respectively, while Gs rose by 23.2%. Under SD treatment, improvements in various indicators were more pronounced: Pn markedly increased 86.2%, and Gs, Tr, and Ci also increased significantly by 45.0%, 29.0%, and 20.2%, respectively.

### 3.4. Effects of S. indica on Cell Membrane Permeability and Reactive Oxygen Species Metabolism in P. sheareri Seedlings Under Drought Stress

**Figure 4:**
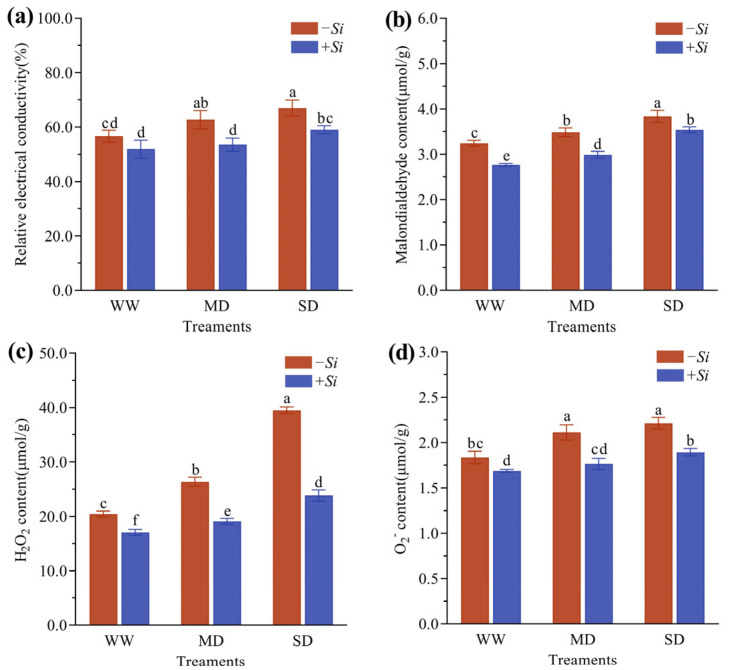
Effects of *S. indica* on plant REC (**a**), MDA content (**b**), H_2_O_2_ content (**c**), and O_2_^−^ content (**d**) under drought stress. The data are presented as the means ± SEs (n = 3); different letters above the bars indicate significant (*p* < 0.05) differences. Abbreviations: WW, well-watered; MD, moderate drought; SD, severe drought; *+Si*, inoculation with *S. indica*; *-Si*, inoculation without *S. indica*.

As drought stress intensified, the relative electrical conductivity (REC), malondiadehyde (MDA), hydrogen peroxide (H_2_O_2_), and superoxide anion (O_2_^−^) content in *P. sheareri* seedlings all exhibited a gradual increasing trend. Under the same drought conditions, the *+Si* treatment significantly reduced all these indicators (Figure 4a–d). Under WW treatment, compared with the *-Si* treatment, the *+Si* treatment significantly decreased REC and MDA by 8.4% and 14.7%, respectively. Under MD treatment, the reductions in REC and MDA expanded to 14.6% and 14.2%. Under SD treatment, significant decreases of 11.9% and 7.8% were still observed.

In terms of reactive oxygen species, the *+Si* treatment also significantly inhibited the accumulation of H_2_O_2_ and O_2_^−^: under WW treatment, they were reduced by 16.5% and 8.0%, respectively; under MD treatment, the decreases reached 27.7% and 16.5%; and under SD treatment, even more pronounced reductions of 39.7% and 14.4% were recorded. These results demonstrate that the *+Si* treatment effectively mitigated membrane lipid peroxidation and oxidative damage induced by MD and SD treatments.

### 3.5. Effects of S. indica on Antioxidant Enzyme Activities in P. sheareri Seedlings Under Drought Stress

**Figure 5:**
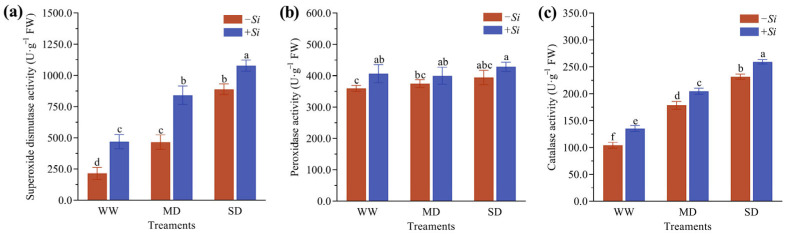
Effects of *S. indica* on SOD (**a**), POD (**b**), and CAT (**c**) Activities in Plants Under Drought Stress. The data are presented as the means ± SEs (n = 3); different letters above the bars indicate significant (*p* < 0.05) differences. Abbreviations: WW, well-watered; MD, moderate drought; SD, severe drought; *+Si*, inoculation with *S. indica*; *-Si*, inoculation without *S. indica*.

As drought stress intensified, the activities of superoxide dismutase (SOD), peroxidase (POD), and catalase (CAT) in *P. sheareri* seedlings all exhibited a gradual increasing trend. The *+Si* treatment further enhanced these antioxidant enzyme activities (Figure 5a–c). Compared with the *-Si* treatment, the *+Si* treatment under WW treatment significantly increased the activities of SOD, POD, and CAT by 118.2%, 13.1%, and 30.1%, respectively. Under MD treatment, the activities of SOD and CAT were significantly enhanced by 80.8% and 14.8%. Even under SD treatment, SOD and CAT activities remained significantly higher than those in the *-Si* treatment by 21.3% and 11.9%, respectively. These results demonstrate that the *+Si* treatment enhanced the antioxidant enzyme activities in *P. sheareri* seedlings, with particularly pronounced effects on SOD and CAT.

### 3.6. Effects of S. indica on Hormone Content in P. sheareri Seedlings Under Drought Stress

**Figure 6:**
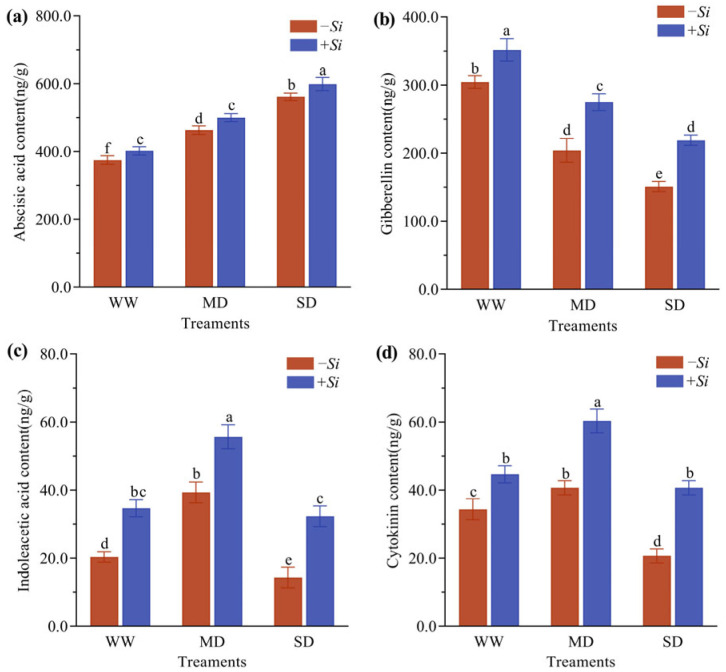
Effects of *S. indica* on Plant ABA (**a**), GA (**b**), IAA (**c**), and CTK (**d**) Content under Drought Stress. The data are presented as the means ± SEs (n = 3); different letters above the bars indicate significant (*p* < 0.05) differences. Abbreviations: WW, well-watered; MD, moderate drought; SD, severe drought; *+Si*, inoculation with *S. indica*; *-Si*, inoculation without *S. indica*.

As drought stress intensified, the abscisic acid (ABA) content in *P. sheareri* seedlings gradually increased, whereas gibberellin (GA) content progressively decreased. The contents of indoleacetic acid (IAA) and cytokinin (CTK) showed an initial increase followed by a decline. Under the same drought conditions, compared with the *-Si* treatment, the *+Si* treatment significantly increased the contents of ABA, GA, IAA, and CTK (Figure 6a–d). Specifically, under WW treatment, the *+Si* treatment significantly increased ABA, GA, IAA, and CTK contents by 6.8%, 15.6%, 70.5%, and 30.6%, respectively, compared to the *-Si* treatment. Under MD treatment, the contents of these four hormones increased by 7.4%, 34.8%, 41.5%, and 48.4%, respectively. Under SD treatment, the *+Si* treatment still led to significant enhancements, with increases of 6.3%, 45.0%, 125.6%, and 96.8%, respectively.

## 4. Discussion

Drought stress generally suppresses overall plant growth, manifested as inhibited shoot morphological development and reduced biomass accumulation, while compelling plants to prioritize the allocation of limited resources to the root system to enhance survival [27]. Previous studies have shown that drought-stressed seedlings of *Juniperus pingii* exhibit reduced plant height, thinner stem diameter, decreased leaf area, and diminished total biomass [28], whereas seedlings of *Sorbus folgneri* under progressive drought displayed adaptive root modifications such as elongation of the primary root and an increased number of lateral roots [29]. Liu et al. [30] also reported that with increasing drought intensity, the height and ground diameter growth of *Pinus yunnanensis* seedlings gradually declined, accompanied by a shift in biomass allocation toward the roots. In this study, *P. sheareri* seedlings under drought stress exhibited typical symptoms including leaf dehydration, wilting, and chlorosis, with the severity increasing under more intense drought conditions; however, inoculation with *S. indica* significantly alleviated these symptoms and mitigated drought-induced damage. It is noteworthy that under moderate drought (MD) conditions, the plants actively adjusted their root architecture by increasing both length and diameter of primary and lateral roots. This response was further enhanced by inoculation with *S. indica*: under both MD and SD treatments, inoculated plants showed significantly increased biomass accumulation and improved root traits—including root length, surface area, and volume—compared to non-inoculated controls, indicating that *S. indica* enhances water uptake capacity and drought survival in *P. sheareri* primarily by promoting root development and expanding the absorptive surface area.

Photosynthesis is one of the physiological processes most significantly affected by drought stress [31]. During the initial phase of drought, plants close their stomata, thereby restricting CO_2_ supply; as the stress intensifies, non-stomatal limitations—including chlorophyll degradation, photosystem damage, and inactivation of key enzymes—become dominant, leading to reduced light-use efficiency and ultimately manifesting as sustained and significant decreases in net photosynthetic rate (Pₙ) and transpiration rate (Tᵣ) [32,33]. Studies have reported consistent declines in Pₙ, Tᵣ, and stomatal conductance (Gₛ) in seedlings of *Xanthoceras sorbifolium* [34] and *Corydalis edulis* [35] under drought conditions. Research on *Aronia melanocarpa* not only observed decreases in Pₙ, Gₛ, and Tᵣ, but also found that photosynthetic pigment content exhibited an initial increase followed by a subsequent decline [36]. In the present study, drought stress significantly reduced both chlorophyll content and photosynthetic parameters in *P. sheareri* seedlings, thereby inhibiting photosynthetic performance. However, inoculation with *S. indica* markedly increased these metrics, indicating that *S. indica* enhances chlorophyll synthesis and subsequently improves photosynthetic efficiency, thereby alleviating drought-induced damage. This finding is consistent with previous research demonstrating that *S. indica* improves drought tolerance in tomato by enhancing photosynthetic and stomatal parameters [37].

Drought stress induces increased oxidative pressure within plant cells, resulting in the excessive production of reactive oxygen species (ROS) such as H_2_O_2_, O_2_^−^, and ·OH [38,39]. These ROS molecules cause oxidative damage to biological macromolecules including cell membranes, proteins, nucleic acids, and chlorophyll, leading to increased membrane permeability and disruption of organelle structure and function [40]. In the present study, drought stress led to substantial accumulation of ROS in *P. sheareri* seedlings, as evidenced by significantly elevated levels of O_2_^−^ and H_2_O_2_. The increases in relative electrical conductivity (REC) and malondialdehyde (MDA) content indicated that the cell membranes of *P. sheareri* seedlings had undergone oxidative damage, which is consistent with the findings reported by Wang et al. regarding the physiological effects of drought stress on *Linum usitatissimum*. In contrast, inoculation with *S. indica* resulted in a reduction in all ROS-related indicators to varying degrees, demonstrating that *S. indica* can mitigate oxidative damage under drought stress by decreasing the accumulation of reactive oxygen species.

When the level of reactive oxygen species (ROS) exceeds the plant’s scavenging capacity, the antioxidant system is activated to maintain cellular redox homeostasis [41]. The coordinated action of key antioxidant enzymes, including superoxide dismutase (SOD), catalase (CAT), and peroxidase (POD), is crucial for scavenging reactive oxygen species (ROS) and thus alleviating oxidative damage in plant cells [42,43]. In this study, the activities of SOD, POD, and CAT increased in *P. sheareri* seedlings under drought stress, indicating an adaptive enzymatic response to mitigate oxidative imbalance. Moreover, inoculation with *S. indica* resulted in a further significant enhancement of these antioxidant enzyme activities, demonstrating that *S. indica* stimulates the host’s antioxidant system, improves ROS-scavenging capacity, and ultimately helps counteract excessive ROS accumulation induced by drought stress. Of particular note, the activities of SOD and CAT were significantly elevated in response to *S. indica* inoculation under both WW and MD regimes relative to the control, pointing to a potential “priming effect” of this fungus on the host’s antioxidant defenses.

Phytohormones, as endogenous signaling molecules, form a complex regulatory network in plant responses to abiotic stress [44]. In this study, drought stress led to a significant increase in ABA and a decrease in GA in *P. sheareri* seedlings, consistent with typical stress responses [45]. Notably, under moderate drought (MD), the contents of IAA and CTK increase significantly, which may lead to root growth and lateral root formation. Inoculation with *S. indica* further elevated the levels of ABA, GA, IAA, and CTK, suggesting that the fungus modulates phytohormone synthesis to promote root development and improve water uptake capacity. These coordinated changes indicate a sophisticated hormonal cross-talk potentially orchestrated by *S. indica*. The concurrent increase in IAA—which promotes lateral root initiation—and CTK—a negative regulator of lateral rooting—implies that the fungus may fine-tune their spatial distribution and signaling sensitivity rather than merely altering bulk concentrations [46,47,48]. This rebalancing likely fosters a root architecture conducive to drought adaptation without suppressing primary growth. Moreover, the sharp rise in ABA, known to interact with auxin transport, may integrate stress signaling with growth promotion, steering root expansion toward moisture-rich zones [49,50]. We therefore propose that *S. indica* acts as a microbial modulator that synchronizes the IAA-CTK-ABA network, pre-adapting the root system for efficient water foraging and enhancing drought tolerance.

## 5. Conclusions

**Figure 7:**
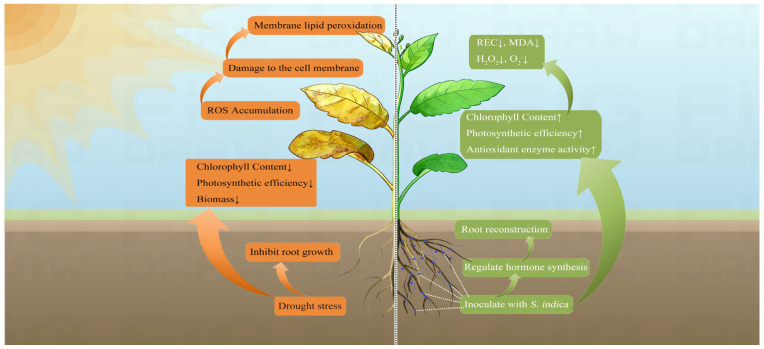
The potential mechanism by which *S. indica* spores enhance the drought resistance of *P. sheareri* seedlings under drought stress. ↑ arrows show an increase, while ↓ arrows show a decrease in the variable.

In this study, drought stress inhibited the growth and photosynthetic capacity of *P. sheareri* seedlings, resulting in substantial accumulation of reactive oxygen species (ROS), which led to cellular membrane damage and membrane lipid peroxidation. Compared with the –*Si* treatment, the +*Si* treatment alleviated drought-induced damage by enhancing photosynthetic efficiency and stimulating the antioxidant system. Furthermore, it promoted root growth through the modulation of hormone synthesis, thereby improving water uptake capacity and enhancing drought tolerance (Figure 7). These findings reveal the responses of *P. sheareri* seedlings to drought stress and further demonstrate the role of *S. indica* in improving plant drought resistance, deepening the theoretical understanding of woody plant–fungal symbiotic interactions. From a practical perspective, inoculating seedlings with *S. indica* shows considerable potential for application in nursery production and afforestation programs, particularly in drought regions. This approach could significantly improve seedling survival rates, thereby offering a viable biological strategy to support ecosystem restoration and the development of climate-resilient forestry.

## Data Availability

The original contributions presented in this study are included in the article. Further inquiries can be directed to the corresponding author.

## References

[1] Takács G., Gergely I., Ördög V., Vörös L., Iváncsics J. (2025). Approaches to studying wheat and maize drought stress responses. Plant Soil.

[2] Verma K.K., Song X.P., Kumari A., Jagadesh M., Singh S.K., Bhatt R., Singh M., Seth C.S., Li Y.R. (2024). Climate change adaptation: Challenges for agricultural sustainability. Plant Cell Environ..

[3] Lima R.P.C., Silva D.D.D.A., Moreira M.C., Passos J.B.M.C., Coelho C.D., Elesbon A.A.A. (2019). Development of an annual drought classification system based on drought severity indexes. An. Da Acad. Bras. De Cienc..

[4] Liu S.X., Ma A.F., Feng Q., Gong Z.Z. (2025). Research advances in plant drought resistance. Chin. Sci. Bull..

[5] Shi F., Pan Z., Dai P., Shen Y., Lu Y., Han B. (2023). Effect of Water logging Stress on Leaf Anatomical Structure and Ultrastructure of *Phoebe sheareri* Seedlings. Forests.

[6] Song Y., Yao X., Tan Y., Gan Y., Yang J., Corlett R.T. (2017). Comparative analysis of complete chloroplast genome sequences of two subtropical trees, *Phoebe sheareri* and *Phoebe omeiensis* (Lauraceae). Tree Genet. Genomes.

[7] Liu H., Liu M., Tang W., Li M.L., Qian Y.Y., Ning L.P. (2017). Wood structural characteristics of *Phoebe sheareri*. J. Northeast For. Univ..

[8] Li J., Chen C., Wen S., Yang L., Sun W., He G., Zhang D. (2024). The cross talk of sesquiterpenes and phenylpropanes mediated by the shikimic acid pathway affects essential oil content in *Phoebe sheareri* leaves. Ind. Crops Prod..

[9] Fan Y., Lou Y.K., Ku W.P., Dai Q.L., Wang Z.Q., Zhao M.S., Yu S.Q. (2020). Age structure and spatial point pattern of *Phoebe sheareri* population in Mount Tianmu. J. Zhejiang AF Univ..

[10] Wang Y., Ma X.H., Lu Y.F., Hu X.E., Lou L.H., Tong Z.K., Zhang J.H. (2022). Assessing the current genetic structure of 21 remnant populations and predicting the impacts of climate change on the geographic distribution of *Phoebe shearer* in southern China. Sci. Total Environ..

[11] Li L., Feng Y., Qi F.Y., Hao R.Y. (2023). Research Progress of Piriformospora indica in Improving Plant Growth and Stress Resistance to Plant. J. Fungi.

[12] Bandyopadhyay P., Yadav B.G., Kumar S.G., Kumar R., Kogel K.H., Kumar S. (2022). *Piriformospora indica* and Azotobacter chroococcum Consortium Facilitates Higher Acquisition of N, P with Improved Carbon Allocation and Enhanced Plant Growth in *Oryza sativa*. J. Fungi.

[13] Shrivastava N., Mahajan S., Jain A., Sharma P., Kharakwal A.C., Varma A. (2019). Mutualistic Interaction of Piriformospora indica (*Serendipita indica*) with Aloe vera, the Wonder Plant for Modern Living. Am. J. Plant Sci..

[14] Das A., Kamal S., Shakil N.A., Sherameti I., Oelmüller R., Dua M., Tuteja N., Johri A.K., Varma A. (2012). The root endophyte fungus *Piriformospora indica* leads to early flowering, higher biomass and altered secondary metabolites of the medicinal plant, *Coleus forskohlii*. Plant Signal. Behav..

[15] Yin L., Qu P.Y., Wang D.M., Yan S.T., Gong Q.H., Yang R., Hu Y., Liu N.R., Cheng C.Z., Wang P.F. (2024). The Influence of *Piriformospora indica* Colonization on the Root Development and Growth of *Cerasus humilis* Cuttings. Plants.

[16] Wang Z.B., Zong F.Q., Lin W., Tang X.X., Xuan S.K., He B.Z., Wu B.H., Guo L.J. (2025). The effects of *Piriformospora indica* on the growth of cuttings from three species of woody ornamental plants. Ind. Crops Prod..

[17] Achatz B., von Rüden S., Andrade D., Neumann E., Pons-Kühnemann J., Kogel K.H., Franken P., Waller F. (2010). Root colonization by *Piriformospora indica* enhances grain yield in barley under diverse nutrient regimes by accelerating plant development. Plant Soil.

[18] Zhu S.Y., Shi F., Li H.H., Ding Y.W., Chang W., Ping Y., Song F.Q. (2024). *Piriformospora indica* alleviates soda saline-alkaline stress in Glycine max by modulating plant metabolism. Front. Plant Sci..

[19] Xu L., Wang A.A., Wang J., Wei Q., Zhang W.Y. (2017). *Piriformospora indica* confers drought tolerance on *Zea may* L. through enhancement of antioxidant activity and expression of drought-related genes. Crop J..

[20] Baltruschat H., Fodor J., Harrach B.D., Niemczyk E., Barna B., Gullner G., Janeczko A., Kogel K.H., Schäfer P., Schwarczinger I. (2008). Salt tolerance of barley induced by the root endophyte Piriformospora indica is associated with a strong increase in antioxidants. New Phytol..

[21] Wu W.J., Liu R.C., Xiao Z.Y., Alqahtani M.D., Wang F.L., Almaabadi A.D., Kuca K., Zou Y.N., Wu Q.S. (2024). Non-targeted metabolomics reveals hormonal mechanisms regarding arbuscular mycorrhizal fungi- and Serendipita indica-mediated plant growth response in Camellia oleifera. Sci. Hortic..

[22] Wei Q., Wu M.Y., Zhang W.Y., Xu L., Chen J.F., Pan R., Tian X.H. (2018). Effect of the endophytic fungus *Piriformospora indica* on the growthand drought tolerance of rice seedling under drought stress. Chin. J. Ecol..

[23] Yang L., Zou Y.N., Tian Z.H., Wu Q.S., Kuca K. (2021). Effects of beneficial endophytic fungal inoculants on plant growth and nutrient absorption of trifoliate orange seedlings. Sci. Hortic..

[24] Barranco D., Ruiz N., Gómez-del Campo M. (2005). Frost tolerance of eight olive cultivars. Hortscience.

[25] Zhou Y., Huang L., Liu S.Y., Zhao M.Y., Liu J.M., Lin L.J., Liu K.D. (2023). Physiological and transcriptomic analysis of IAA-induced antioxidant defense and cell wall metabolism in postharvest mango fruit. Food Res. Int..

[26] Liu R.C., Gao W.Q., Srivastava A.K., Zou Y.N., Kuca K., Hashem A., Abd-Allah E.F., Wu Q.S. (2021). Differential Effects of Exogenous Glomalin-Related Soil Proteins on Plant Growth of Trifoliate Orange Through Regulating Auxin Changes. Front. Plant Sci..

[27] Guo C.C., Bao X.Y., Sun H.C., Zhu L.X., Zhang Y.J., Zhang K., Bai Z.Y., Zhu J.J., Liu X.Q., Li A.C. (2024). Optimizing root system architecture to improve cotton drought tolerance and minimize yield loss during mild drought stress. Field Crops Res..

[28] Zhuoma L., Xin F.H., Yang X.L., Zhao K.T. (2015). Effect of drought stress on growth and physiological indicators of *Sabina pingii* var.wilsonii seedlings. J. Northwest AF Univ. (Nat. Sci. Ed.).

[29] Chen X., Xu Y.F. (2011). Effects of drought stress on growth and physiological characteristics in Sorbus folgneri seedlings. J. For. Environ..

[30] Liu C.Y., Liang S.Y., Wu J.W., Gu J.Y., Duan H.J. (2026). Response of growth and physiological-biochemical characteristics in *Pinus yunnanensis* seedlings to drought and rewatering. J. Northwest AF Univ. (Nat. Sci. Ed.).

[31] Qiao M.Y., Hong C.H., Jiao Y.J., Hou S.J., Gao H.B. (2024). Impacts of Drought on Photosynthesis in Major Food Crops and the Related Mechanisms of Plant Responses to Drought. Plants.

[32] Lin S., Zhang W.M., Wang G.F., Hu Y.X., Zhong X.B., Tang G.X. (2024). Physiological Regulation of Photosynthetic-Related Indices, Antioxidant Defense, and Proline Anabolism on Drought Tolerance of Wild Soybean (*Glycine soja* L.). Plants.

[33] Tian Q.F., Zhang H.C., Bian L.M., Zhou L., Ge Y.F., Casella E. (2024). Three-Dimensional Quantification and Visualization of Leaf Chlorophyll Content in Poplar Saplings under Drought Using SFM-MVS. Forests.

[34] Zong J.W., Chang Y.W., Zhu Y.Q., Deng H.F., Cai Y.Y., Yang Y.H. (2025). Effects of AM fungi on *Xanthoceras sorbifolium* growth and photosynthetic physiology under drought stress. J. Northwest AF Univ. (Nat. Sci. Ed.).

[35] Dai W.J., Ding Y.F., Zhao Y., Zhou Y., Fan H.Y. (2025). Effects of drought stress on physiological and photosyntheticcharacteristics of three species of *Corydalis*. J. Northwest AF Univ. (Nat. Sci. Ed.).

[36] Maimaiti A., Tuohetimaimaiti Z., Jiang Y., Qin Q., Nur Y., Xu M. (2024). Photosynthetic and physiological responses of Aronia Melanocarpa seedlings to soil drought. Non-Wood For. Res..

[37] Miranda V., Silva-Castro G.A., Ruiz-Lozano J.M., Fracchia S., García-Romera I. (2023). Fungal Endophytes Enhance Wheat and Tomato Drought Tolerance in Terms of Plant Growth and Biochemical Parameters. J. Fungi.

[38] Baxter A., Mittler R., Suzuki N. (2014). ROS as key players in plant stress signalling. J. Exp. Bot..

[39] Mittler R., Zandalinas S.I., Fichman Y., Van Breusegem F. (2022). Reactive oxygen species signalling in plant stress responses. Nat. Rev. Mol. Cell Biol..

[40] Miller G., Suzuki N., Ciftci-Yilmaz S., Mittler R. (2010). Reactive oxygen species homeostasis and signalling during drought and salinity stresses. Plant Cell Environ..

[41] Wang P.T., Liu W.C., Han C., Wang S.T., Bai M.Y., Song C.P. (2024). Reactive oxygen species: Multidimensional regulators of plant adaptation to abiotic stress and development. J. Integr. Plant Biol..

[42] Luo D.L., Zhai G.F., Cao S., Zhu T., Chen R.J., Xiao Q., Zhang D., Ba L.J. (2022). Effect of preharvest salicylic acid combined with postharvest 1-MCP treatment on quality and antioxidant ability of plum fruit during storage. Sci. Technol. Food Ind..

[43] Ma J.J., Bai M.H., Paizilijiang O., Tian R., Tang P.L., Song Y.N., Jieken G., Cui K.B. (2025). Effects of ozone treatment on AsA-GSH cycle and membrane lipid peroxidation in postharvest storage of prunus fruits. Sci. Technol. Food Ind..

[44] Jiang Y., Huang J.S., Xu Y.M., Chen Y.T., Xie B., Hu J. (2025). Antagonistic mechanisms of phytohormones ABA and GA in plant droughtresponses. Biotic Resour..

[45] Vidal A., Cantabella D., Bernal-Vicente A., Díaz-Vivancos P., Hernández J.A. (2018). Nitrate- and nitric oxide-induced plant growth in pea seedlings is linked to antioxidative metabolism and the ABA/GA balance. J. Plant Physiol..

[46] Liao Z.Q., Chen B.B., Boubakri H., Farooq M., Mur L.A.J., Urano D., Teo C.H., Tan B.C., Hasan M.M., Aslam M.M. (2025). The regulatory role of phytohormones in plant drought tolerance. Planta.

[47] Xie Z.Z., Jin L., Sun Y., Zhan C.H., Tang S.Q., Qin T., Liu N., Huang J.L. (2024). OsNAC120 balances plant growth and drought tolerance by integrating GA and ABA signaling in rice. Plant Commun..

[48] Zhang Y.Z., Li Y.P., Hassan M.J., Li Z., Peng Y. (2020). Indole-3-acetic acid improves drought tolerance of white clover via activating auxin, abscisic acid and jasmonic acid related genes and inhibiting senescence genes. BMC Plant Biol..

[49] Zhang Y.Z., Qin X.F., He Z.R., Zhang Y., Li Z., Nie G., Zhao J.M., Feng G.Y., Peng Y. (2024). The White Clover TrMYB33-TrSAMS1 Module Contributes to Drought Tolerance by Modulation of Spermidine Biosynthesis via an ABA-Dependent Pathway. Int. J. Mol. Sci..

[50] Li J.J., Li Y., Yin Z.G., Jiang J.H., Zhang M.H., Guo X., Ye Z.J., Zhao Y., Xiong H.Y., Zhang Z.Y. (2017). OsASR5 enhances drought tolerance through a stomatal closure pathway associated with ABA and H2O2 signalling in rice. Plant Biotechnol. J..

